# Antibacterial Effect and Therapy of Chronic Skin Defects Using the Composite Bioscaffold Polycaprolactone/GelitaSpon/Povidone-Iodine in Domestic Dogs

**DOI:** 10.3390/polym15214201

**Published:** 2023-10-24

**Authors:** Barbora Šišková, Martin Kožár, Radka Staroňová, Ivan Shepa, Vanda Hajdučková, Patrícia Hudecová, Michaela Kaduková, Marek Schnitzer

**Affiliations:** 1 Small Animal Clinic, University of Veterinary Medicine and Pharmacy, Komenského 73, 041 81 Košice, Slovakia; barbora.siskova@student.uvlf.sk (B.Š.); radka.staronova@uvlf.sk (R.S.); 2 Institute of Material Research, Slovak Academy of Sciences, 040 01 Košice, Slovakia; ishepa@saske.sk; 3 Department of Microbiology and Immunology, University of Veterinary Medicine and Pharmacy, Komenského 73, 041 81 Košice, Slovakia; vanda.hajduckova@uvlf.sk (V.H.); patricia.hudecova@student.uvlf.sk (P.H.); 4 Department of Parasitology, University of Veterinary Medicine and Pharmacy, Komenského 73, 041 81 Košice, Slovakia; michaela.kadukova@student.uvlf.sk; 5 Faculty of Mechanical Engineering, Department of Biomedical Engineering and Measurement, Technical University of Košice, Letná 9, 042 00 Košice, Slovakia; marek.schnitzer@tuke.sk

**Keywords:** chronic wound, gelatin, iodine, PCL, wound healing

## Abstract

Chronic wounds and the failure of conventional treatment are relatively common in veterinary medicine. Recently, there has been a growing interest in alternative therapeutic approaches and the utilization of biodegradable materials. Their potential application in wound therapy may offer a novel and more suitable option compared to conventional treatment methods. Biodegradable materials can be classified into two main categories: natural, synthetic, and a combination of both, which have the potential to have synergistically enhanced properties. In this study, four domestic dogs with clinical symptoms of chronic wounds were enrolled. These wounds underwent treatment utilizing a novel biodegradable composite material composed of gelatin sponge combined with two electrospun layers of polycaprolactone (PCL) along with polyvinylpyrrolidone (PVP) fibers containing povidone-iodine complex (PVP-I). The initial phase of the study was dedicated to evaluating the antibacterial properties of iodine against *Staphylococcus aureus* and *Escherichia coli*. On average, wound healing in domestic dogs took 22 days from the initial treatment, and iodine concentrations demonstrated a significant antibacterial effect against *Escherichia coli* and *Staphylococcus aureus*. Based on the favorable outcomes observed in wound management, we believe that the utilization of a blend of natural and synthetic biodegradable materials holds promise as an effective wound therapy option.

## 1. Introduction

Wounds, categorized as acute or chronic, based on their healing process and duration, represent an interruption in skin or mucosal continuity [1]. Acute wounds progress through distinct phases—hemostasis, inflammation, proliferation, and remodeling—leading to the restoration of structural integrity. Chronic wounds, characterized by persistent inflammation, infections, and necrosis, typically fail to heal within three months [2,3]. Various factors, such as necrotic tissue, pathogen contamination, and underlying conditions like diabetes, can impede the healing process, thereby extending each phase [4].

Effective wound closure aims to swiftly restore anatomical and functional integrity through surgical reconstruction and medical treatments [5,6]. Effective care for chronic wounds requires a multimodal approach, including wound bed optimization, management of chronic medical conditions, and consistent monitoring [7]. Traditional wound dressings such as cotton and wool are being gradually replaced by advanced materials that create a protective environment and facilitate the transfer of active substances [4]. 

Nanotechnological approaches applied to biomaterials recently have gained increased attention thanks to their potential in the treatment of chronic wounds [8]. These materials include sponges, hydrogels, films, and hydrofiber mats, incorporating synthetic and natural elements [4].

Electrospun nanofibers, known for their highly porous structure resembling the extracellular matrix, support cell adhesion, proliferation, differentiation, and therapeutic agent delivery—this is why they gained special interest and wide application in tissue engineering [9,10,11].

Gelatin—an irreversibly hydrolyzed form of collagen, holds promise in nanopharmaceuticals and drug delivery due to its biodegradability and high biocompatibility [12]. The use of gelatin has gained popularity for several reasons: it is more readily available, highly soluble, and it is much cheaper compared to other ECM proteins and thus is easier to use for biomedical purposes. Gelatin possesses a highly similar structure to collagen and contains important binding moieties for cell attachment. Gelatin obtained from different sources (fish skin, porcine skin, etc.) is biocompatible, biodegradable, and does not induce antigenicity and toxicity in cells [13,14]. It has been used in medical and pharmaceutical fields as a matrix for implants and as stabilizers in vaccines such as measles, mumps, and rubella [15]. Moreover, there are forms of gelatin that are water-permeable and soluble in water and have multifunctional properties, such as a drug-delivery carrier [16].

While gelatin has its merits, it also presents challenges, like poor mechanical properties, lack of thermal stability, and a relatively shorter degradation rate [13]. These limitations can be mitigated through gelatin modification and the formation of gelatin-based composites to enhance mechanical stability, biocompatibility, and bioactivity [17]. 

Polycaprolactone is one of the most regularly used synthetic polymers for medical applications thanks to its slow, controllable biodegradation and biocompatible characteristics [18]. Due to its biodegradability, it is used in the preparation of scaffolds for wound repair and bone tissue regeneration [19,20,21]. Polycaprolactone has been extensively researched due to its peculiar mechanical properties, as well as its miscibility with several polymers [22]. Its slow degradation rate and hydrophobic nature make it suitable for long-term implants and drug-delivery systems [23,24]. Electrospun PCL membranes can be either used as skin substitutes or as wound dressings, allowing for the incorporation of bioactive substances [25]. 

Polycaprolactone is a synthetic polymer with good spinnability and mechanical properties; however, it has shortcomings, such as a slow degradation rate and deprived hydrophilicity [26,27]. Therefore, a combination of gelatin and PCL could be applied to produce a composite that overcomes the disadvantages of each [28]. 

In the pursuit of novel wound management solutions, this study explores the potential of PCL/GelitaSpon/PVP-I as a promising novel wound dressing material. The hypothesis driving this research is that the combination of GelitaSpon, a biodegradable commercially available and widely tested sponge, PCL, and PVP-I will lead to accelerated wound healing in dogs compared to conventional wound management techniques. Our research aims to investigate whether this composite material can accelerate wound healing compared to conventional techniques. Additionally, we assess the antibacterial properties of PVP-I integrated into the dressing material and closely monitor any potential complications or side effects arising from its use.

## 2. Materials and Methods

### 2.1. Domestic Dogs

Four domestic dogs with chronic non-healing skin defects were referred to the Small Animal Clinic of the University Veterinary Hospital, University of Veterinary Medicine and Pharmacy in Košice, Slovakia. The experimental study included domestic dogs for which conventional wound therapy had failed, leading to the application of alternative treatment. The dogs in the study were mixed breeds (*n* = 3) and one Rhodesian Ridgeback (*n* = 1), consisting of three females and one male. The experimental study encompassed wounds of various etiologies, such as wound dehiscence (*n* = 2), a puncture wound (*n* = 1), and a laceration wound (*n* = 1) located on different parts of the body.

**Figure 1:**
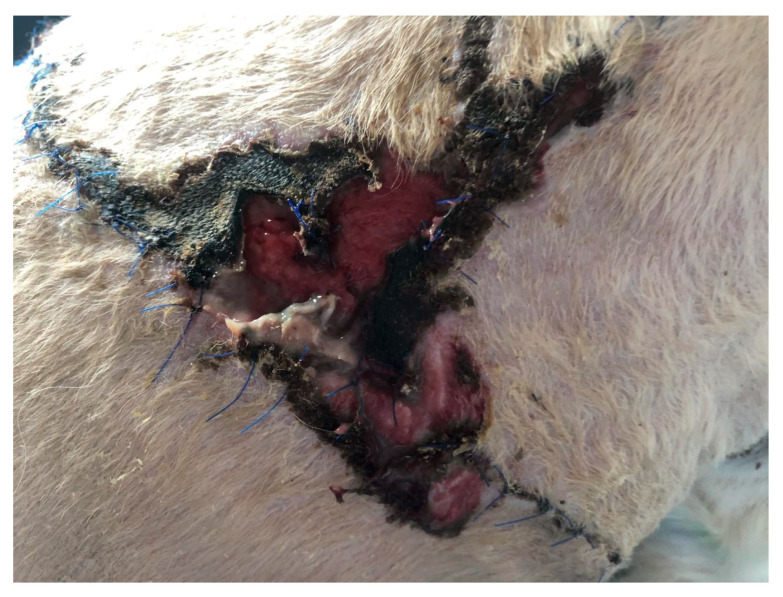
Wound of D1 with a massive amount of necrotic tissue.

In the first case (D1), a dog, a male crossbreed six years old, weighing 23 kg, was referred to the clinic with a wound dehiscence following a bite injury. The wound exhibited an irregular shape, measuring approximately 25 × 14 cm, and was situated in the dorsal region on the right side of the thoracic cavity. Within the wound, a significant amount of necrotic tissue, secernation, and surface drying was evident (Figure 1). After surgical debridement to remove necrotic tissue, a prepared biodegradable material, PCL/GelitaSpon/PVP-I, was applied to the wound surface in combination with topical ointments containing antibiotics (ATB).

**Figure 2:**
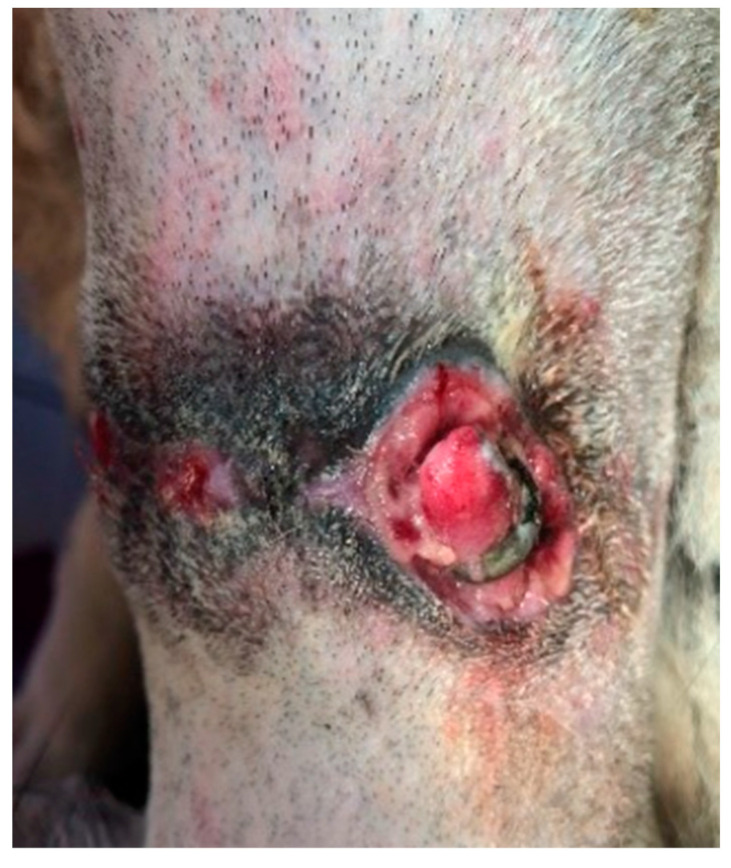
Round-shaped injury localized on the dorsal surface of the neck (D2).

The second domestic dog (D2), a one-year-old female crossbreed weighing 25 kg, displayed remarkably swift wound closure. This dog had a chronic wound that extended across 2/3 of the dorsal surface of the neck, a result of a chain becoming embedded in the neck (Figure 2). To treat this wound, PCL/GelitaSpon/PVP-I was applied to the wound surface. Additionally, a thin layer of ointment containing ATB was used in combination with the biodegradable material.

**Figure 3:**
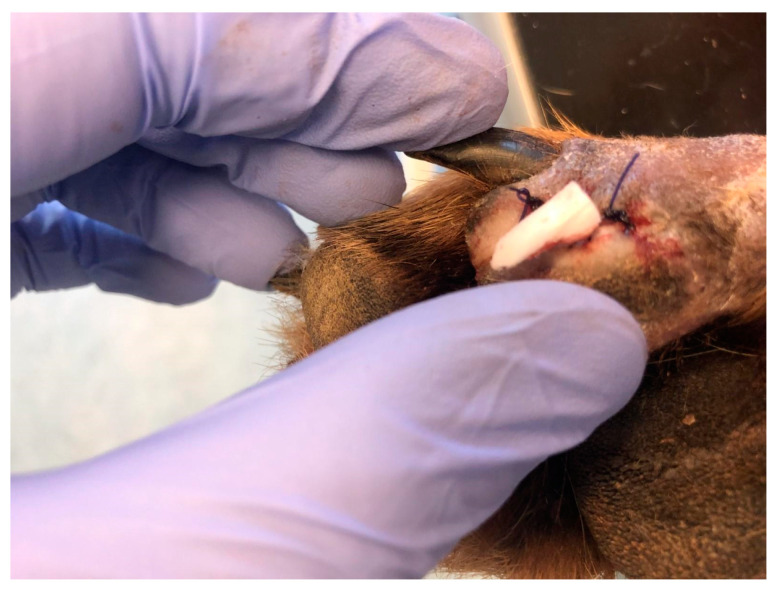
The round-shaped wound on the plantar surface of the pad of D3 with an application of PCL/GelitaSpon/PVP-I.

The third domestic dog (D3), an eight-year-old female Rhodesian Ridgeback weighing 45 kg, displayed the shortest treatment duration. This dog was diagnosed with a circular stab wound measuring 2 cm in diameter on the plantar pad of the fifth toe of the left pelvic limb. The wound displayed hypergranulation, significant swelling, and active infection at the injury site. To address this, PCL/GelitaSpon/PVP-I was applied to the wound surface, along with a thin layer of ATB-containing ointment (Figure 3).

**Figure 4:**
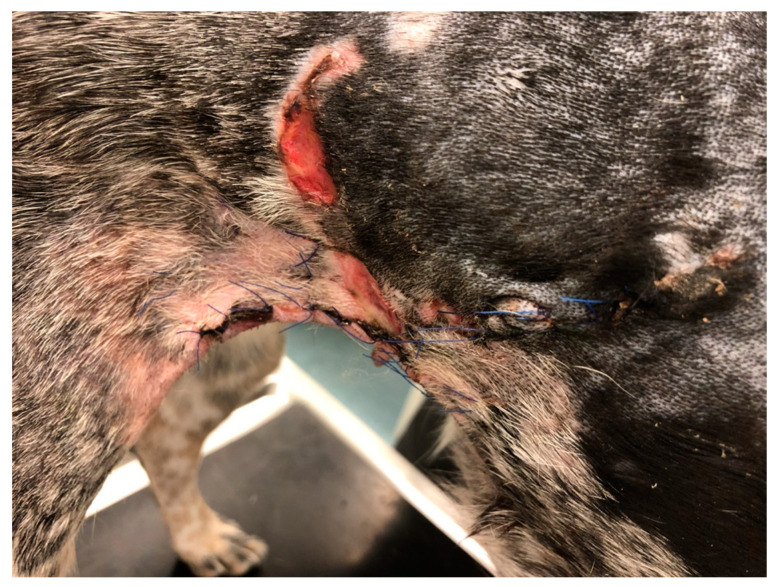
Dehiscence of a wound in the area of the right groin (D4).

The last case (D4), a five-year-old female of mixed breed weighing 14 kg, was brought to the clinic due to the dehiscence of a surgically treated laceration wound. This wound was situated in the middle of the right side of the abdominal wall, extending into the area of the right groin (Figure 4). To address this issue, the wound site was thoroughly cleaned of surface contaminants, and PCL/GelitaSpon/PVP-I, in conjunction with ointments containing ATB, was applied to the wound site.

The dogs included in our study were referred to our facility from other clinics after the failure of traditional therapy methods. In the case of the first domestic dog (D1), surgical closure of the bite wound was initially chosen, but wound dehiscence with necrosis of superficial structures occurred. Subsequently, conservative therapeutic management with the application of topical ointments to the wound surface was chosen. However, no visible improvement in the healing process was monitored for over two weeks. As a result, the patient was referred to our clinic. For the second dog (D2), primary conservative wound management with the application of local ointments to the wound surface was chosen for a period of two weeks, but similarly to D1, no progress in the healing process was registered. Consequently, the dog was referred to our clinic. Similarly, the third dog, D3, received primary conservative wound management with the application of local ointments to the wound surface for three weeks, but there was no improvement in the healing process, leading to the referral of this dog to our clinic and further inclusion in this study. In the case of the fourth dog, D4, surgical primary closure of the laceration was initially chosen, but wound dehiscence occurred, prompting the referral of the dog to our clinic.

D1–D4 dogs received alternative wound management with the use of biodegradable composite materials. These dogs serve as their own control group because the initial therapy choice failed. It is not possible to create a control group for these dogs referred to the clinic, as is possible in laboratory conditions where a uniform skin defect can be created, because each dog presents a different type of injury, localization, extent, and overall factors that affect the healing process, such as age and health status.

### 2.2. Preparation of Biodegradable Material—PCL/GelitaSpon/PVP-I 

Polyvinylpyrrolidone (PVP, Acros Organics, Geel, Belgium, M_w_ = 50,000 g.mol^−1^), polycaprolactone (PCL, M_w_ = 80,000 g.mol^−1^ Aldrich, Burlington, MA, USA), formic acid (MICROCHEM, Pezinok, Slovakia, anhydrous), and acetic acid (MICROCHEM, Pezinok, Slovakia, glacial) were used without additional purification for the preparation of the solution for needleless electrospinning.

GelitaSpon Standart GS-002 (GELITA MEDICAL GmbH, Eberbach, Germany) and Braunoderm^®^ (B. Braun Melsungen AG, Melsungen, Germany) were obtained from the local drug store. GelitaSpon Standart GS-002, further referred to as GelitaSpon, is an absorbable gelatin sponge used as a hemostatic. In comparison, Braunoderm^®^ is a commercial alcohol-based povidone-iodine complex (PVP-I) solution for skin disinfection before surgical procedures and was used as a source of the PVP-I. The amount of active ingredients per 100 g solution is 50.0 g propane-2-ol (isopropyl alcohol) and 1 g povidone-iodine (with 10 wt.% available iodine). Pure PVP polymer was used as a polymer carrier, mandatory for the fiber formation during the electrospinning. Prepared PVP fibers containing PVP-I, further referred to as PVP-I fibers or PVP-I fibrous mat. The concentration of the PVP-I complex in the solution is 1 wt.%. The initial mass ratio of PVP/Braunoderm^®^/acetic acid, used for the preparation of the electrospinning solution, was 15/45/2, which refers to the 25 wt.% of the PVP polymer in the solution. Acetic acid was added to modify the conductivity and surface tension of the solution and evaporated during the electrospinning process. The concentration of the PVP-I complex in the final fibrous product is 2.90 wt.% due to the removal of the volatile species. 

The PVP-I solution for electrospinning was prepared as follows: the appropriate amount of PVP polymer was dissolved in the Braunoderm^®^ liquid and mixed at 25 °C by a magnetic stirrer for one hour. Then, 3.2 wt.% of acetic acid was added to the precursor solution and further mixed for an hour.

The PCL solution with a concentration of 10 wt.% was prepared by dissolving PCL granules in pure formic acid and mixing with a magnetic stirrer at 80 °C for 2 h. Before the electrospinning, the solution was cooled to room temperature and electrospun only as a fresh one (within 2 h after full PCL dissolution). 

The as-prepared solutions were electrospun at the same conditions from a rotating wireframe electrode of the Nanospider^TM^ NS Lab200 machine from ELMARCO Co. (Liberec, Czech Republic), using a needle-less electrospinning technology. The applied voltage was 82 kV, the distance from the electrode to the collector was 150 mm, and the electrode rotation rate—6 rpm. Electrospinning was performed at ambient temperature (25 °C) with a relative humidity of 50%. 

The commercial sponge GelitaSpon (GELITA MEDICAL GmbH, Eberbach, Germany) was removed from its package and fixed by sterile single-use transdermal needles on the PP (polypropylene) spunbond substrate, commonly used for electrospinning, and placed in the Nanospider machine. The sandwich structure preparation started with the deposition of the PVP-I fibrous layer. After 20 min of electrospinning, the spunbond, together with the sponges, was removed from the deposition chamber. Then, the sponges were removed with prior cutting out from the excessive fibrous mat. The mat can be used separately to prepare a sandwich with non-connected layers or locally on wounds as a source of iodine. After the separation of the sponges from the PVP-I fibrous mat, the sponges were flipped on the other side, fixed in the same manner on the new piece of spun-bond, and placed inside the electrospinning machine for the deposition of the PCL layer. The deposition of PCL was performed at the same conditions and deposition time. The prepared sandwiches were stored in sterile Petri dishes, and all the tools used were sterilized by 96 wt.% ethanol and/or 254 nm UVC lamp.

The morphology of the prepared samples was observed by scanning electron microscopy (Dual-beam SEM/FIB ZEISS AURIGA Compact equipped with EDX, Carl Zeiss, Oberkochen, Germany) and optical microscopy (Axio Observer D1M, inverted optical microscope, Carl Zeiss, Oberkochen, Germany).

The preparation of sandwiches (including solutions and the electrospinning itself) and the scanning electron microscopy of the fibrous mats were performed at the Laboratory of Nanotechnology and SEM/FIB Laboratory of the Institute of Materials Research of Slovak Academy of Sciences. 

### 2.3. Antibacterial Activity of PVP-I Tested Spectrophotometrically

Bacterial strains Escherichia coli CCM 3954 and Staphylococcus aureus CCM 4223 (Czech Collection of Microorganisms, Brno) were employed for testing the antibacterial activity of PVP-I. The antibacterial effect of PVP-I corresponds to iodine at a concentration of 10 wt.% in a 100 g solution. Iodine’s antibacterial activity was determined spectrophotometrically by measuring absorbance. Various concentrations of iodine, including 4 wt.%, 2 wt.%, 1 wt.%, 0.5 wt.%, 0.25 wt.%, and 0.125 wt.%, were prepared by dilution in Brain-Heart Infusion (BHI) broth within 96-well plates. To create a 1.0 McFarland scale suspension, an 18 h culture of the tested strains was prepared in a sterile physiological solution. The prepared bacterial strain suspensions were added to the diluted iodine in a 1:1 ratio. After 24 h of incubation at 37 °C, the antibacterial activity of iodine was determined using spectrophotometric absorbance measurements at a wavelength of 600 nm using a Biotek Synergy 2 instrument. BHI broth with the tested strain was used as a control. The results were evaluated using Dunnett’s test in the statistical software Prism 8.3.0.

### 2.4. Wound Management 

Upon the admission of each domestic dog to the clinic, their overall condition was assessed. Subsequently, a macroscopic evaluation of the wound was performed, considering its localization, extent, secernation, hyperemia, edema, and surface necrosis. These macroscopic indicators were evaluated during each bandage change. Based on the changes in these individual macroscopic indicators, the wound treatment strategy was adjusted. This underlines that, in clinical practice, it is not feasible to treat every wound uniformly, even within the various phases of the wound healing process. Therefore, it is imperative to tailor the treatment for each dog’s specific wound condition.

The next step involved sample collection, which was then sent for microbiological cultivation at a certified laboratory. Systemic antibiotic treatment was determined based on the results of antibacterial sensitivity testing. According to the results, a combination of amoxicillin with clavulanic acid (Synulox, Zoetis Czech republic s.r.o., Prague, Czech Republic) was administered at a dose of 15 mg·kg–1 b.w. twice daily per os, and enrofloxacin (Enroxil, KRKA, d.d., Novo Mesto, Slovenia) was given at a dose of 5 mg·kg^−1^ b.w. of body weight once daily per os.

Following the sample collection for microbial culturing, the wound bed was prepared. If necrotic tissue was present in the wound, surgical debridement was performed. Subsequently, the wound was cleansed of contaminants using a bacteriostatic and bactericidal lavage solution (Braunol, B. Braun Melsungen AG, Melsungen, Germany) diluted in an isotonic solution (0.9 wt.% NaCl, B. Braun Melsungen AG, Melsungen, Germany).

A specially prepared biodegradable material, PCL/GelitaSpon/PVP-I, was applied to the wound surface. In areas where the wound extended beyond the coverage of the biodegradable material, preparations in the form of creams and ointments were used. These included a cream containing silver sulfadiazine and sodium hyaluronate (Ialugen plus, IBSA, Slovakia s.r.o., Bratislava, Slovakia), an ointment containing neomycin and bacitracin (Baneocin, Sandoz GmbH, Holzkirchen, Austria), and an iodine-containing ointment (Betadine, EGIS Pharmaceuticals PLC, Budapest, Hungary). For surface coverage, a dressing composed of three layers was employed: a primary layer of gauze squares (Lohmann and Rauscher, Rengsdorf, Germany), a secondary layer of synthetic cotton known as Cellona^®^ (Lohmann and Rauscher, Rengsdorf, Germany), and an elastic CoPoly dressing (CoPoly, M+H VET s.r.o., Prague, Czech Republic) applied in a circular manner over the wound site.

Wound assessments were conducted individually for each domestic dog on different days, considering the stage and progress of wound healing. This approach was essential as each domestic dog required an individualized approach, and it was not feasible to propose a uniform wound therapy plan based on the dynamics of the wound-healing process in each dog.

## 3. Results

### 3.1. Optical and SEM Micrographs of the Prepared Samples

**Figure 5:**
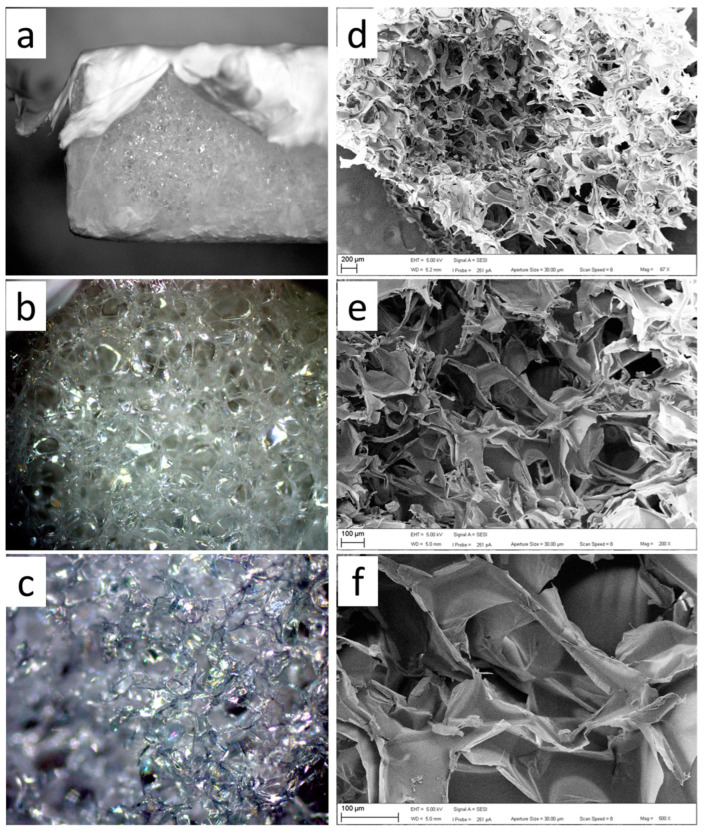
Optical (**a**–**c**) and SEM (**e**,**f**) micrographs of the GelitaSpon sponge captured at different magnifications: (**a**) 8×; (**b**) 25×; (**c**) 50×; (**d**) 67×; (**e**) 200×; and (**f**) 500×.

**Figure 6:**
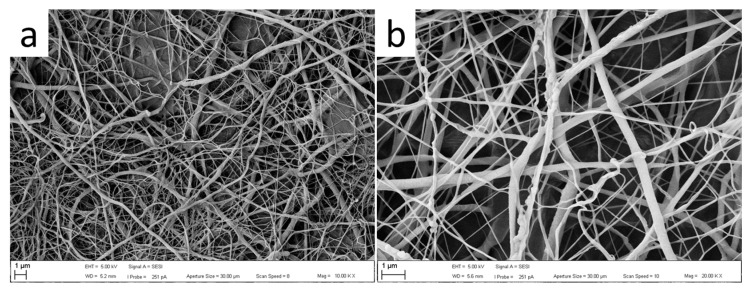
SEM micrographs of the PCL nanofibers at different magnifications: (**a**) 2 kx; (**b**) 20 kx.

**Figure 7:**
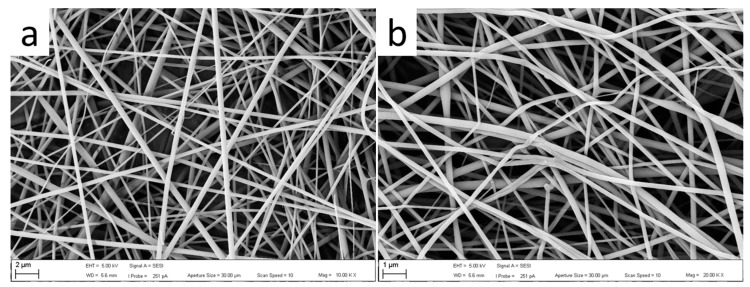
SEM micrographs of the PVP-I nanofibers at different magnifications: (**a**) 10 Kx; (**b**) 20 Kx.

Optical and SEM micrographs of the commercial GelitaSpon sponge used are presented in Figure 5a, where layers of PVP-I fibers (upper) and PCL (lower) are visible. SEM micrographs of the prepared electrospun PCL and PVP-I are presented in Figure 6 and Figure 7, respectively.

### 3.2. Antibacterial Activity of PVP-I Tested Spectrophotometrically 

**Figure 8:**
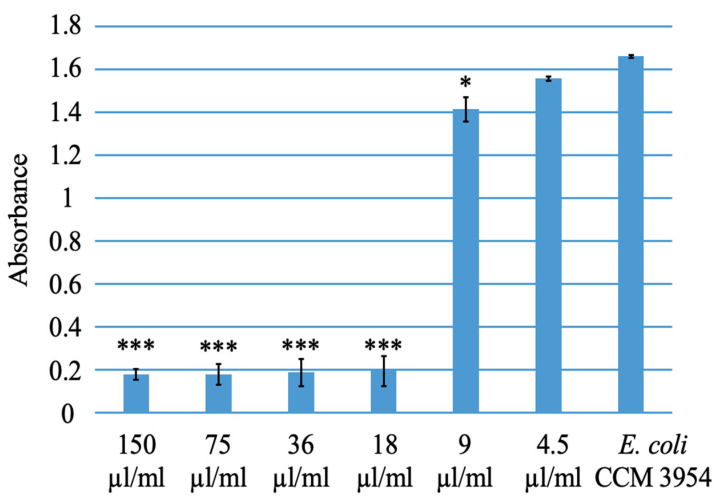
Testing the antibacterial activity of PVP-I against *Escherichia coli* CCM 3954. * = *p* value adjusted < 0.05, *** = *p* value adjusted < 0.001.

**Figure 9:**
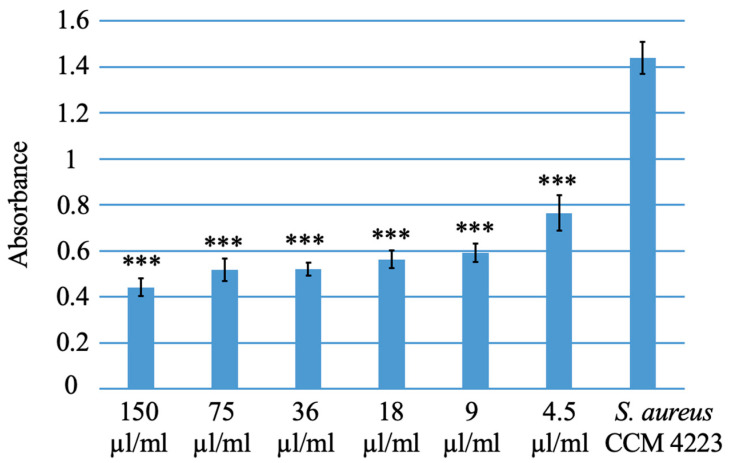
Testing the antibacterial activity of PVP-I against *Staphylococcus aureus* CCM 4223. *** = *p* value adjusted < 0.001.

The antibacterial activity of PVP-I against *Escherichia coli* CCM 3954 and *Staphylococcus aureus* CCM 4223 was tested spectrophotometrically using a serial dilution method in microtiter plates, measuring absorbance with a Biotek Synergy 2 instrument. All used iodine concentrations exhibited significant antibacterial effects against *Escherichia coli* CCM 3954 compared to the control (Figure 8) and also against *Staphylococcus aureus* CCM 4223 (Figure 9).

### 3.3. Domestic Dogs 

The average healing time for domestic dogs from the primary treatment and application of PCL/GelitaSpon/PVP-I covering to complete closure of the defect was 22 days. The shortest healing period observed from the initial treatment was 14 days, while the longest duration of therapy required from the initial treatment was 38 days. We selected this combination because gelatin, except for its primary hemostatic effect, acts as a biocompatible scaffold, offering structural support and facilitating cell adhesion, proliferation, and migration. Simultaneously, PCL and PVP fibers serve as controlled-release carriers for therapeutic substances, delivering essential growth factors and antimicrobials, such as PVP-I in this case, directly to the wound site. Polycaprolactone gradually degrades over time, ensuring sustained support throughout the healing process and reducing the necessity for frequent dressings removal and changes.

We monitored fundamental macroscopic parameters in all domestic dogs, serving as the basis for wound assessment. These parameters included secernation, hyperemia, edema, and necrotization of the defect surface. All these indicators indicated the transition of the wounds to the chronic phase, characterized by a stagnant formation of granulation tissue.

Initially, all dogs exhibited varying degrees of hyperemia, which subsided during the course of therapy. Wound secretion was observed in two dogs but also improved as the therapy progressed. In the first dog, D1, a substantial amount of necrotic tissue was present in the wound, necessitating surgical debridement. The swelling was a common feature in all dogs, albeit to varying extents.

The presence of granulation tissue emerged as a crucial marker; most defects lacked granulation tissue, signaling their transition to chronicity. The use of biodegradable materials facilitated progress in the healing process, leading to the closure of wounds in the shortest possible time, restoration of damaged tissue functionality, and the achievement of a satisfactory cosmetic outcome.

**Figure 10:**
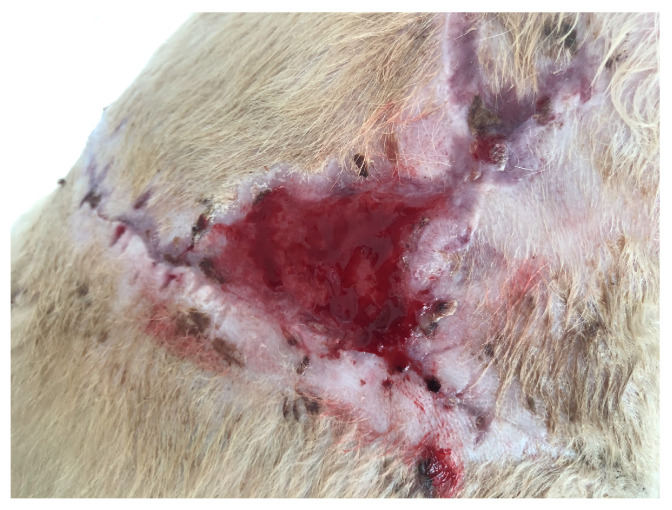
The wound of D1 on the third day after the start of therapy had detached necrotic tissue and formed healthy granulation tissue with an epithelialization lining at the edge of the wound.

**Figure 11:**
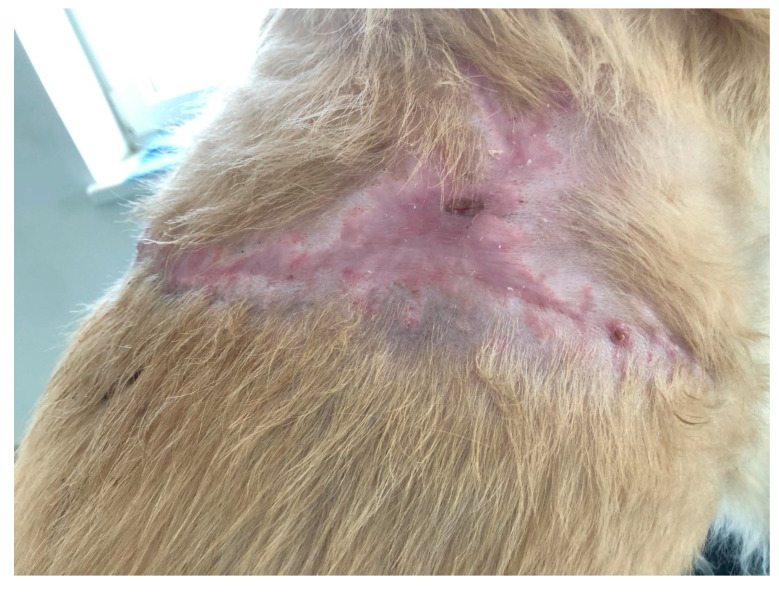
Closure of the defect on day 38 from the start of therapy (D1).

In the case of D1, with surgical wound dehiscence resulting from a bite injury, we observed the formation of healthy granulation tissue and the development of an epithelialization lining at the wound’s edges on the third day of treatment (Figure 10). Treatment was sustained until the skin defect completely closed, which occurred on the 38th day from the initiation of treatment (Figure 11).

**Figure 12:**
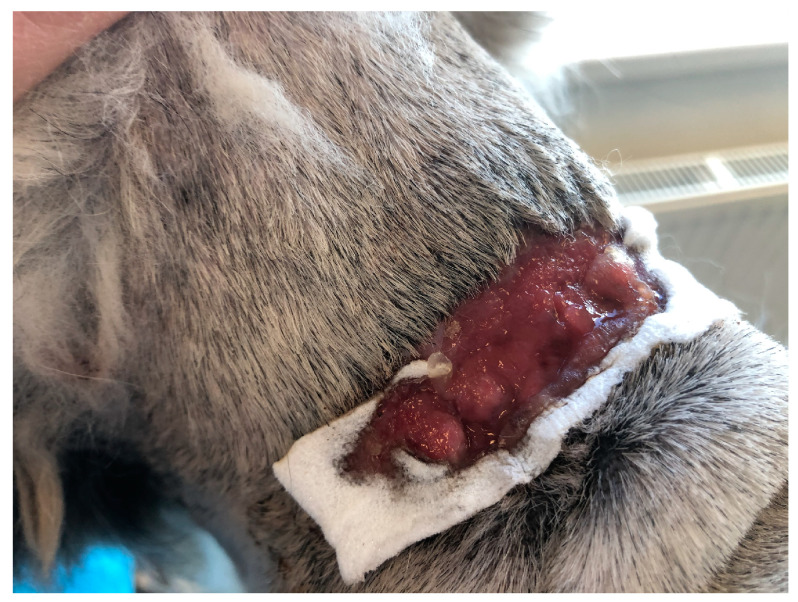
The wound of D2, 72 h after initiation of therapy, formed healthy granulation tissue and debrided necrotic tissue.

**Figure 13:**
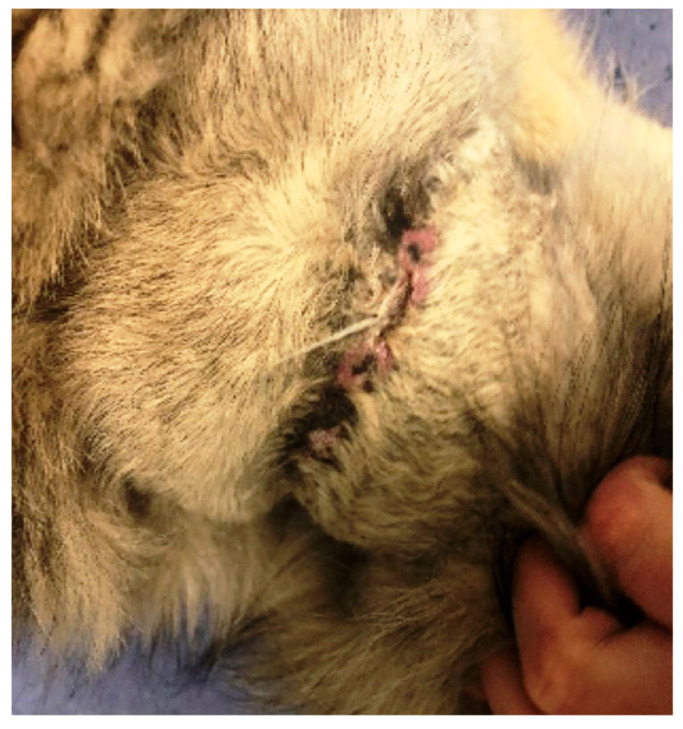
Closure of the defect on day 15 from the start of therapy (D2).

In the case of D2, with a chronic wound on the dorsal surface of the neck, necrotic tissue was eliminated through enzymatic debridement within the first 72 h of treatment (Figure 12). The wound was successfully closed by the 15th day from the outset of treatment (Figure 13).

**Figure 14:**
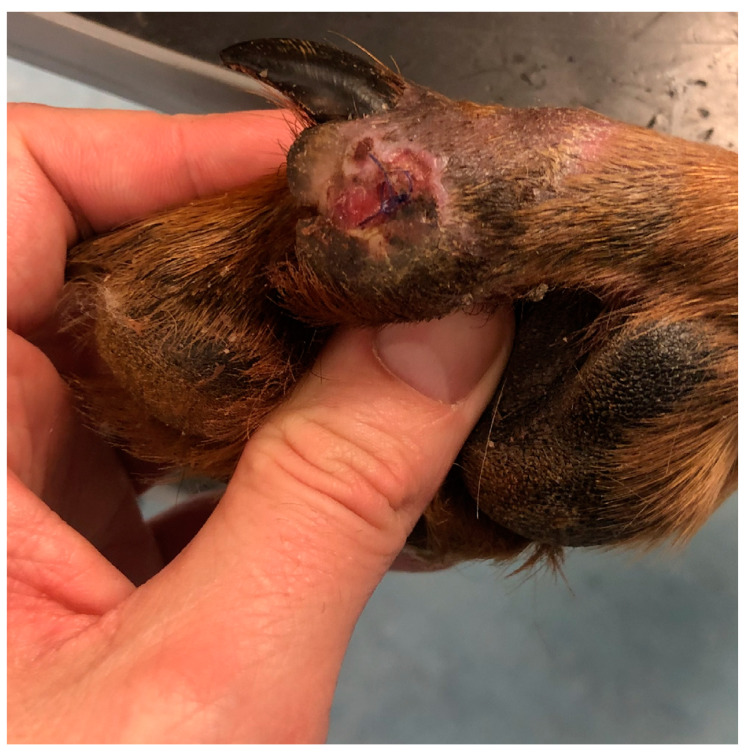
Closure of the defect on day 11 from the start of therapy in D3.

**Figure 15:**
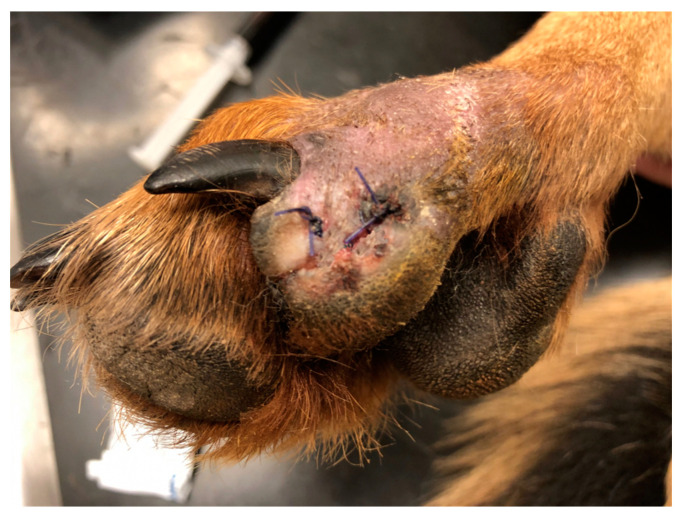
Reepithelization of the defect on day 14 from the start of therapy (D3).

The third dog, D3, which had a stab wound on the plantar surface of the fifth toe of the left pelvic limb, experienced a reduction in swelling within the initial 48 h. Significant formation of new granulation tissue followed after debridement of the affected areas. The wound successfully closed 11 days after the initiation of treatment (Figure 14). Reepithelialization of the wound surface was observed on the 14th day following the initiation of treatment (Figure 15).

**Figure 16:**
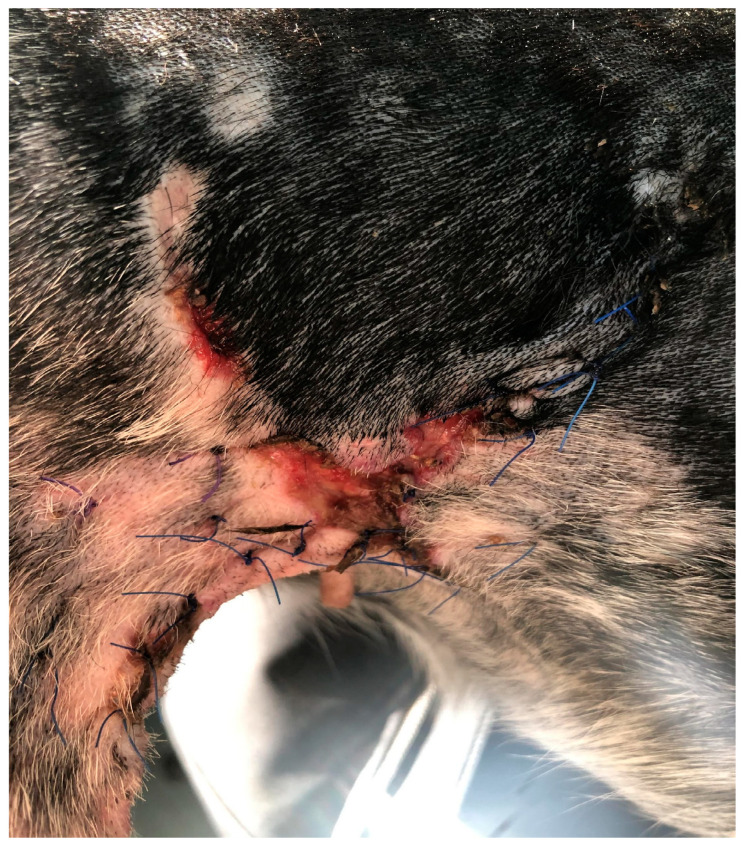
Appearance of the wound of D4 14 days after the start of therapy.

**Figure 17:**
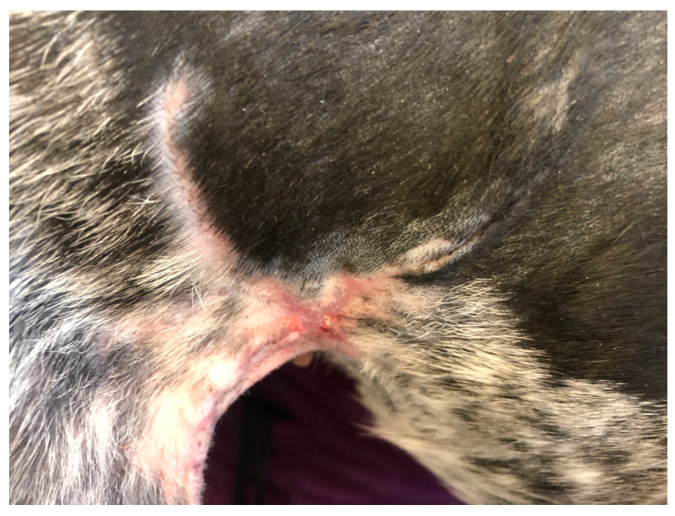
Closure of the defect on day 21 from the start of therapy (D4).

Regarding the D4 with a wound in the groin area, there was a notable decrease in swelling and wound discharge just four days after the treatment commenced. By the 14th day of treatment, the wound had shrunk by 90 % in size (Figure 16), and complete wound healing was observed by the 21st day following the initiation of treatment (Figure 17).

Our findings demonstrate that the blend of natural and synthetic materials effectively facilitates the healing process in dogs without causing any complications during their recovery.

## 4. Discussion

Iodine is the heaviest essential element required by all living organisms. Molecular iodine possesses potent antimicrobial properties and has long been used as a skin disinfectant [29,30]. Nevertheless, its practicality is hampered by its tendency to rapidly evaporate, limiting its effectiveness in clinical applications. To address this limitation, researchers have sought to harness the antimicrobial potential of iodine by incorporating it into functionalized polymers [31]. Scientific studies have increasingly explored the use of both natural and synthetic polymers in creating polymer-iodine complexes [32,33,34]. In a study by Tang et al., chitosan-based iodine complexes were investigated. Chitosan, a widely used natural polysaccharide, is favored for its non-toxic, biocompatible, biodegradable, and antimicrobial properties. The research found that the chitosan-iodine complex demonstrated significant antibacterial activity against *E. coli* and *S. aureus* [35]. Gao and colleagues examined the antimicrobial effects of povidone-iodine nanoparticles (povidone-iodine NPs) against Escherichia coli, Staphylococcus aureus, and Pseudomonas aeruginosa. They discovered that after incorporating povidone-iodine NPs, products such as adhesives, ink, and dye exhibited substantial antibacterial properties [36]. Takaya et al. observed that by applying 0.1 wt.% iodine through electrodeposition on aluminum after anodization, the modified metal displayed strong antibacterial activity against *S. aureus* and *E. coli* [37]. In a separate study, Roberts and their team tested various concentrations of povidone-iodine for disinfecting eyelids and ocular surfaces in dogs. They recommended a povidone-iodine dilution ratio of 1:50 for disinfecting ocular surfaces in pre-surgical procedures [38]. While these studies offer valuable insights into iodine-based antimicrobial materials, it is crucial to acknowledge that the clinical translation of such materials may face challenges related to sustained efficacy and patient-specific variations in wound conditions.

Researchers have devoted significant attention to studying effective wound dressing and scaffolding techniques for promoting healing over the years. Among the various biomaterials investigated, gelatin and PCL have stood out due to their distinctive properties and promising outcomes in the context of wound healing applications. Various studies have showcased their unique properties and potential in wound healing applications. In a study by Liu et al., gelatin-based wound dressings were evaluated in a rat model of full-thickness skin wounds. The results demonstrated profound wound healing, characterized by accelerated reepithelialization, increased collagen deposition, and reduced scar formation. The authors also emphasized that the incorporation of other materials can have synergistic effects and thus can improve the wound-healing process [39]. Furthermore, Yao et al. developed a gelatin bilayer scaffold containing an extract of Lithospermi radix (LR) that was successfully slowly released from the nanofibers, as well as conducting new tissue regeneration and wound recovery in rats. Remarkably, the gelatin not only demonstrated non-cytotoxic effects but also supported cell attachment to the scaffold. The incorporation of LR further amplified the wound-healing capabilities and bolstered the potential of these gelatin-based scaffolds [40]. Salehi et al. conducted a study focusing on the impact of nanofibrous PCL/gelatin pads infused with varying concentrations of cinnamon extract in the healing process of back wounds in rats. Notably, the most favorable outcome was observed with the PCL/gelatin pad containing 5 % of the extract. In this group, complete closure of the wound defect was achieved within 14 days, while other groups exhibited a small percentage of unhealed defects [41]. In a study conducted by Fallah et al., the research focused on the impact of an antibacterial composite material consisting of gelatin and PCL enriched with curcumin in the wound healing process. Notably, the nanofibers exhibited a remarkable antibacterial activity of 99.9 % against methicillin-resistant Staphylococcus aureus (MRSA) and 82.56 % activity against broad-spectrum beta-lactamases. These compelling results suggest that hybrid nanofibers containing curcumin have significant potential for application in the treatment of infected wounds [42]. In a study conducted by Jiang et al., the researchers explored the impact of PCL/gelatin nanofibrous support enriched with palmatine. In vitro experiments demonstrated significant antioxidant and antibacterial activity. Furthermore, in vivo and histological studies conducted in a rabbit model revealed that the palmatine-containing PCL/gelatin nanofibers not only accelerated the healing process but also effectively prevented the development of hypertrophic scars. These scaffolds demonstrated the ability to facilitate the adhesion, spreading, and proliferation of fibroblasts, showcasing their promising biocompatibility and potential for wound healing applications [28,43]. Farzamfar et al. conducted a study in rats to assess the effectiveness of gelatin/PCL coverage enriched with taurine. The results indicated that in the group treated with nanofibers, there was a remarkable 92 % reduction in the defect size after 14 days, whereas the control group exhibited a 68 % reduction. These findings underscore the potential benefits of incorporating taurine into gelatin/PCL materials for enhancing wound healing [44]. Unalan et al., in their study, investigated the antibacterial effect of gelatin/PCL material containing clove extract. Their antimicrobial investigations revealed significant inhibitory effects against both *Escherichia coli* and *Staphylococcus aureus* [45]. In a study conducted by Sowmya et al., they explored the potential of PCL-based electrospun nanofibrous scaffolds for wound healing applications. The results demonstrated accelerated wound closure, increased collagen production, and enhanced vascularization compared to control groups. Furthermore, these PCL scaffolds effectively facilitated the migration and proliferation of fibroblasts, which are key cells involved in wound healing and provided a three-dimensional matrix for tissue regeneration, impressively similar to the extracellular matrix [46]. 

While these studies collectively indicate the substantial therapeutic promise of gelatin and PCL-based materials for wound healing, it is essential to critically assess their advantages and disadvantages. Advantages encompass their biocompatibility, ability to support tissue regeneration, and potential for controlled release of bioactive compounds. One of the key advantages of the materials is their biodegradability. They can gradually break down in the body, reducing the need for secondary removal procedures. These materials enable the controlled release of therapeutic agents, such as antimicrobial compounds or growth factors, directly to the wound site. Gelatin and PCL possess mechanical properties that can provide structural support to the wound site. This is crucial for preventing contracture and promoting tissue regeneration.

Nevertheless, challenges such as a mismatch in the material degradation time compared to the healing process, variable responses in different wound environments, and potential immunological reactions must be considered. Additionally, the practical application of these materials in clinical settings should be evaluated, accounting for factors like ease of use, patient comfort, and cost-effectiveness.

Gelatin and PCL materials degrade over time. This degradation rate may not always align with the required wound healing timeline. For instance, in chronic wounds or wounds with extended healing processes, the rapid degradation of these materials may limit their effectiveness. Conversely, in acute wounds that heal quickly, the residual presence of these materials after healing could lead to complications. Patients’ unique physiological conditions and genetic factors can influence the performance of biodegradable materials. Factors such as age, underlying health conditions, and genetic predispositions can affect how the body interacts with these materials.

Despite the potential concerns mentioned earlier that could arise when utilizing these materials, the findings from these studies underline the considerable therapeutic potential of gelatin and PCL as wound-healing materials. As ongoing research delves deeper into understanding the interactions between these materials and biological systems, it paves the way for the development of innovative wound treatment strategies. These discoveries hold the promise of transforming the field of wound care and improving the quality of life for individuals facing chronic or severe wounds.

## 5. Conclusions

The utilization of gelatin combined with PCL and PVP-I nanofibers in the treatment of chronic wounds has demonstrated remarkable effectiveness, instilling optimism for enhanced outcomes in dogs and improved processes for healing chronic wounds. Extensive research has revealed that the combination of gelatin and PCL creates a synergistic effect, fostering accelerated tissue regeneration and wound closure while restoring the functionality of damaged tissues and achieving a satisfactory cosmetic result. Moreover, the proposed gelatin and PCL combination showcases exceptional biocompatibility and biodegradability, thus minimizing the risk of adverse reactions during the dog’s recovery. In conclusion, the described PCL/GelitaSpon/PVP-I composite in the treatment of chronic wounds represents a novel and potent approach to addressing the intricate challenges posed by large, non-healing chronic wounds. It showed to be a promising avenue for veterinarians, offering tailored treatment options that stimulate healing, alleviate dog discomfort, and ultimately enhance their quality of life.

## Data Availability

The data presented in this study are available on request from the corresponding author.
